# Supplementary figures and images for: Predicting the impacts of sea level rise in sea turtle nesting habitat on Bioko Island, Equatorial Guinea
Source: PLoS One. 2020 Jul 29;15(7):e0222251. doi: 10.1371/journal.pone.0222251 (PMC7390326; doi:10.1371/journal.pone.0222251)

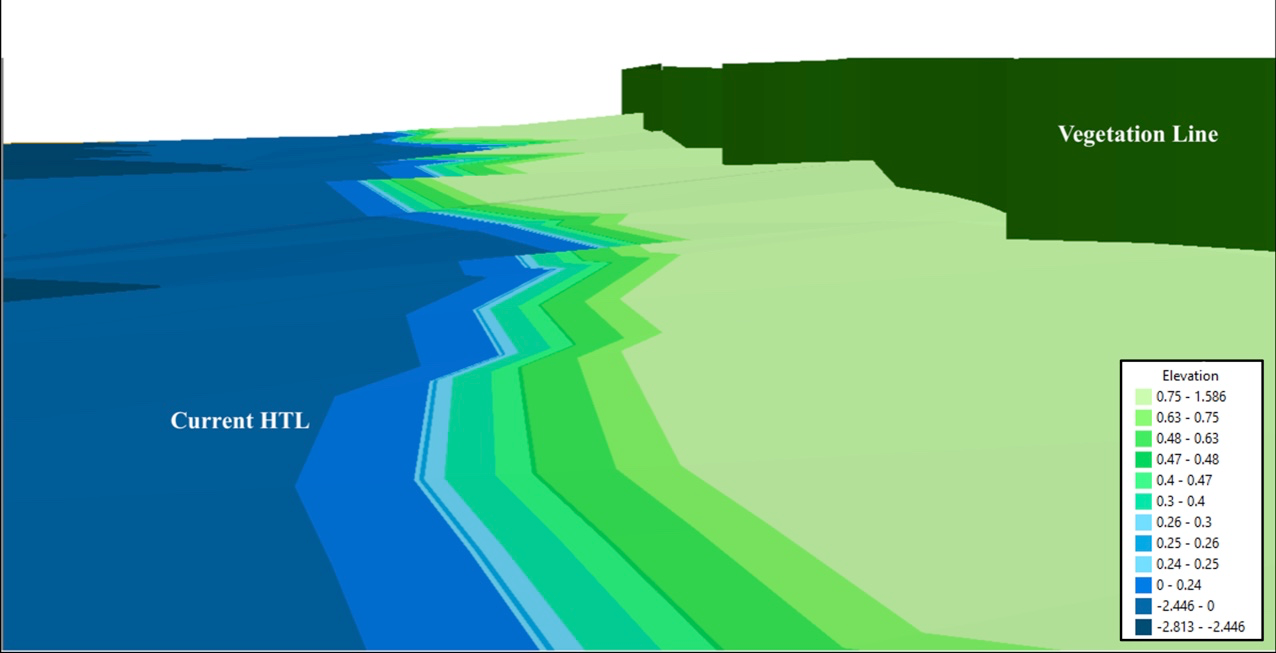

Supplement: S1 Fig — A screenshot of the triangulated irregular network model for Beach D. Displayed are the projected increases in sea level predicted for 2046–2065 scenarios of 0.24, 0.25, 0.26, and 0.3 m and the 2081–2100 scenarios of 0.47, 0.48, 0.63, and 0.75 m. (PNG) [file pone.0222251.s002.png]

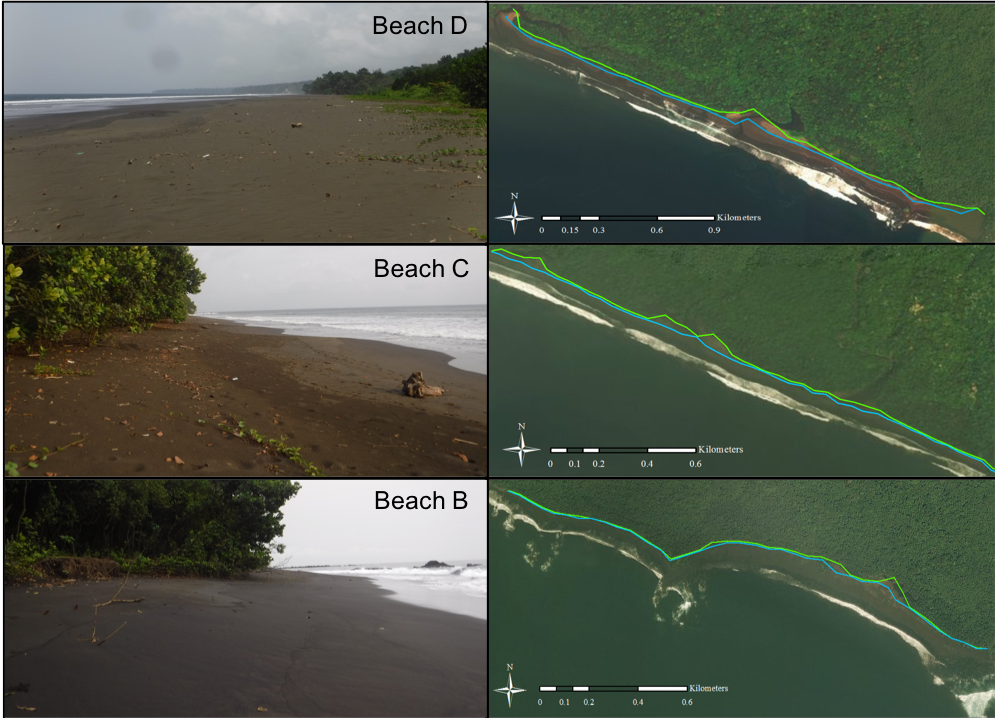

Supplement: S2 Fig — This figure illustrates the visible space in between the HTL and vegetation line, the current nesting habitat, on Beaches D, C and B. HTL shown in blue and vegetation line shown in green. (PNG) [file pone.0222251.s003.png]
